# Spatial organization and early signaling of the B-cell receptor in CLL

**DOI:** 10.3389/fimmu.2022.953660

**Published:** 2022-08-09

**Authors:** Yamit Shorer Arbel, Yotam Bronstein, Tali Dadosh, Talia Kamdjou, Shlomo Tsuriel, Mika Shapiro, Ben-Zion Katz, Yair Herishanu

**Affiliations:** ^1^ Sackler Faculty of Medicine, Tel Aviv University, Tel-Aviv, Israel; ^2^ Department of Hematology, Tel Aviv Sourasky Medical Center, Tel Aviv, Israel; ^3^ Department of Chemical Research Support, Weizmann Institute of Science, Rehovot, Israel; ^4^ Department of Pathology, Tel Aviv Sourasky Medical Center, Tel Aviv, Israel

**Keywords:** CLL (chronic lymphocytic leukemia), BCR signaling, IGHV mutational status, IgM, IgG, d-STORM

## Abstract

Most chronic lymphocytic leukemia (CLL) clones express B-cell receptors (BcR) of both IgM/IgD isotypes; however, 5%–10% of CLL cases express isotype-switched immunoglobulin G (IgG). The early signaling and spatial patterning of the various BcRs at steady state and after activation are still fully unresolved. Herein, we show higher expression of the BcR signalosome elements and a more robust constitutive cell-intrinsic proximal BcR signaling in CLL with unmutated IGHV expressing IgM isotype (IgM U-CLL), compared with IGHV-mutated CLL (M-CLL) expressing either IgM or IgG isotypes. IgM in U-CLL is frequently located in the membrane plane in polarized patches, occasionally in caps, and sometimes inside the cells. Among M-CLL, IgM is scattered laterally in the membrane plane in a similar pattern as seen in normal B cells, whereas IgG is dispersed around the cell membrane in smaller clusters than in IgM U-CLL. Upon BcR engagement, both IgG and IgM expressing M-CLL showed attenuated signaling and only slight spatial reorganization dynamics of BcR microclusters and internalization, compared with the extensive reorganization and internalization of the BcR in IgM expressing U-CLL. The global gene signature of IgG M-CLL was closely related to that of IgM M-CLL rather than IgM U-CLL. Overall, we report fundamental differences in the basal composition, biochemical status, and spatial organization of the BcR in the three examined immunogenetic CLL subtypes that correlate with their clinical behavior. On the basis of our findings, IgG class-switched M-CLL likely represents the same disease as IgM M-CLL rather than a different biological and/or clinical entity.

## Introduction

Chronic lymphocytic leukemia (CLL) is a hematologic malignancy characterized by the progressive accumulation of monoclonal, mature-appearing CD5^+^ B cells in the peripheral blood, bone marrow, and secondary lymphoid organs (1). In recent years, the understanding of the biology of the disease has led to the development of effective treatments, but none of them is curative.

B-cell receptor (BcR) signaling has a critical role in the pathogenesis of CLL (2–6). This is amply attested by the distinct outcomes of patients with different configuration of the clonotypic BcR immunoglobulin (IG). In fact, CLL is dichotomized into two groups based on the somatic hypermutation (SHM) status of the IGHV gene. IGHV-mutated CLL (M-CLL) is a slower progressive disease, whereas IGHV-unmutated CLL (U-CLL) is more aggressive (7, 8). Most CLL clones express BcR of both IgM and IgD isotypes. However, a small proportion of cases, around 5%–10%, express isotype-switched IgGs (9, 10). Isotype-switched IgG^+^ CLL almost exclusively belong to the M-CLL subset (11). IgG^+^ CLL is phenotypically similar to IgM/IgD CLL (12), with a highly skewed immunoglobulin heavy variable (IGHV) gene repertoire that is distinct from IgM^+^ CLL and overuses the IGHV4-34 and IGHV4-39 genes (11). The BcR responsiveness of CLL cells is heterogeneous (13). U-CLL cells are typically BcR competent, whereas M-CLL cells are often anergic (6). BcRs in CLL cells were found to induce cell-autonomous signaling through inter-molecular interactions (14). The strength of these BcR–BcR interactions was shown to correlate with the clinical course of the disease, with high affinity being associated with indolent disease and weaker with an aggressive disease course (15). Considering the essential role of BcR signaling in CLL pathogenesis, unsurprisingly, this pathway has become a target for anti-CLL therapy. Small molecules directed against kinases of the BcR pathway such as Bruton’s tyrosine kinase (BTK) inhibitors show impressive clinical activity in CLL. Nevertheless, because of relapses and transformation, CLL is still considered incurable (4, 16).

Normally, BcR activation is initiated by antigen-induced receptor self-clustering, followed by interactions of the BcR with lipid rafts and lipid raft-resident kinases, including Lyn (17, 18). Lyn phosphorylates the immunoreceptor tyrosine-based activation motifs (ITAMs) on the Igα/Igβ (CD79a/CD79b) subunits of the BcR, leading to recruitment of signaling molecules (e.g., Syk, BLNK, BTK, and PLCγ2), thereby forming microsignalosomes that initiate downstream signaling (18, 19). To form receptor self-clustering, the BcRs must overcome the diffusion barrier within the membrane plane, created by the cortical actin cytoskeleton. The diffusion of the BcR complex within the membrane plane is critical for further activation of the receptor and for the initiation of downstream signaling responses (18, 20, 21). Activation of the BcR affects multiple downstream effectors, which are differentially activated, depending on the state of cell maturation and antigen ligation. These downstream effectors include the mitogen-activated protein kinase (MEK)/extracellular signal-regulated kinase (ERK), phosphatidylinositol 3-kinase(PI3K)/Akt, and nuclear factor-kappa B (NF-kB) pathways, and others. Dysregulation of the BcR signaling pathway in CLL is characterized by constitutively active phosphorylation of certain kinases, leading to cell survival and proliferation (16).

Although the role of the BcR in the pathogenesis of CLL is well established, the early signaling and spatial patterning of the BcR at steady state and after activation, specifically in the context of the IGHV subtypes and immunoglobulin subclasses are still not fully resolved. Herein, we show that IgM U-CLL exhibits a more robust constitutive cell-intrinsic proximal BcR signaling compared with M-CLL expressing either IgM (IgM M-CLL) or IgG (IgG M-CLL). The BcRs in resting IgM U-CLL are commonly organized in polarized large microclusters forming patches and occasionally caps. The IgM BcRs in M-CLL are uniformly distributed across the cell membrane, whereas the IgGs are more diffusely microclustered. Upon BcR engagement, both IgG M and IgM M-CLL trigger poorer signaling and display only slight spatial reorganization of the BcR microclusters and internalization, compared with IgM U-CLL. The gene expression signature of IgM and IgG M-CLL appears to be closely related (albeit with some notable confirmed differences), and both are associated with favorable clinical outcome unlike the aggressive IgM U-CLL subtype.

## Methods

### Patients and samples

Cells were obtained from peripheral blood samples donated by patients fulfilling the standard criteria for CLL after signing informed consent approved by the Tel-Aviv Sourasky Medical’s Institutional Review Board according to the Helsinki Accords. Peripheral blood mononuclear cells (PBMCs) were isolated by Ficoll density gradient centrifugation. Viable frozen cells were kept in fetal calf serum (FCS) containing 10% DMSO and stored in liquid nitrogen. Before use, frozen cells were thawed and cultured at 37°C, 5% CO_2_, in RPMI medium supplemented with 10% FCS, penicillin-streptomycin, and L-glutamine. The samples used contained more than 90% CLL cells. Patient characteristics are presented in **Supplementary Table 1**.

### Antibodies and reagents

ERK1/2, phospho-ERK1/2 (Thr202/Tyr204), Akt (pan), phospho-Akt (S473), CD79b, CD79a, phospho-CD79a (Tyr182), SHP1, phospho-SHP1 (Tyr564), SHIP1, phospho-SHIP1 (Tyr1020), BTK, phospho-BTK(Tyr223), NF-kB p65, phospho–NF-kB p65 (ser536), IgM, Lyn, phospho-Lyn (Y507), LCK, CD86, and ZAP70 antibodies were from Cell Signaling Technology (Beverly, MA). Anti-SRC family (phospho-Y418)–phospho-Lyn (Y396), CD62L, and IgG antibodies were from Abcam (Cambridge, UK). Purified anti-human actin antibody was obtained from MP Biomedicals (Illkirch, France). Goat anti Rabbit IgG (H+L)–HRP conjugate and Goat anti Mouse IgG (H+L)–HRP conjugate, Goat F(ab′)2 anti-human IgM and Goat F(ab′)2 anti-human IgG were from Jackson Immunoresearch Laboratories (West Grove, PA). All antibodies utilized in the study were used in concentrations according to the manufacturer’s instructions. Ficoll-Paque PLUS from GE healthcare (Uppsala, Sweden), dimethyl sulfoxide (DMSO) from Merck (Darmstadt, Germany), RPMI, fetal calf serum (FCS), Dulbecco’s phosphate-buffered saline (PBS), L-glutamine, and penicillin-streptomycin from Biological Industries (Beit-Haemek, Israel) were utilized for cell cultures.

### CD19^+^ cell enrichment

PBMCs were magnetically labeled using CD19 microbeads (Miltenyi Biotec, Inc., Auburn, CA, USA) and separated (more than 95% purity) on a magnetic cell separation LS column (Miltenyi Biotec, Inc.) according to the manufacturer’s instructions.

### Western blotting

CLL cells were lysed in RIPA lysis buffer (Cell Signaling Technology, Beverly, MA) containing phosphatase inhibitor cocktail 2 and protease inhibitor cocktail (Sigma-Aldrich, MO, USA). Extract from cell lysates were separated on 4%–15% Criterion™ TGX™ Precast Midi Protein Gel (Bio-Rad Laboratories) and transferred electrophoretically to nitrocellulose membrane (Bio-Rad Laboratories). The membranes were incubated with the designated antibodies and HRP-conjugated secondary antibodies according to the manufacturer’s instructions. Bands were detected using MyECL Imager (Thermo Scientific, Rockford, IL).

### Immunofluorescence

CLL cells (20 × 10^6^/ml) were seeded on poly-L-lysine–coated glass and then left either inactivated or activated with F(ab′)2 Fragment anti-IgM or F(ab′)2 Fragment anti-IgG at 10 µg/ml (Jackson ImmunoResearch, PA, USA) for 5, 15, and 40 min. The cells were fixed with 4% methanol-free paraformaldehyde for 20 min, followed by permeabilization with 0.2% TX-100 (Sigma Aldrich, MO, USA). The antibodies used were fluorescein isothiocyanate (FITC)-conjugated anti-IgM, FITC-conjugated anti-IgG (Bethyl, TX, USA) (1:200) for 60 min, and DyLight 594 Phalloidin (Cell Signaling Technology, MA, USA) (1:20) for 20 min. Phospho-CD79a (Tyr182) (1:200) and anti-rabbit IgG (H+L), F(ab′)2 Fragment (Alexa Fluor^®^ 594 Conjugate) (1:500), Cell Signaling Technology (Beverly, MA), were used for pCD79a staining. LAMP1 (1:100) and anti-mouse IgG (H+L), F(ab′)2 Fragment (Alexa Fluor^®^ 594 Conjugate) (1:500), were used for LAMP1 staining. After incubation, cells were washed three times with 1% bovine serum albumin (BSA) in PBS. All the cells were labeled with fluorescent mounting medium with DAPI (Golden Bridge International, Inc., WA, USA). Images were acquired using Leica SP8 confocal microscope (Sackler Faculty of Medicine core facility, Tel Aviv University). Quantification was performed using Imaris (version 9.3.1) software.

### Three-dimensional super-resolution microscopy (d-STORM)

Peripheral blood CLL cells were cultured in six-well dishes (20 × 10^6^ cells/ml in RPMI 10% FCS). After 24 h of incubation, cells were fixed with 4% methanol-free PFA and 0.2% glutaraldehyde at room temperature for 20 min, followed by two washes in PBS to remove any excess of fixative. Then, cells were permeabilized with 0.2% TX-100 (Sigma, MO, USA) for 5 min. For blocking, cells were incubated with 3% BSA + 0.2% TX-100 in PBS for 30 min. Then, cells were incubated 60 min with Alexa Fluor^®^ 647-AffiniPure Fab Fragment Goat Anti-Human IgM or IgG (1:100) (Jackson Immunoresearch Laboratories, West Grove, PA) diluted in 1% BSA + 0.2% TX-100 in PBS. After incubation, cells were washed three times with PBS and were dropped on poly-L-lysine–coated Mattek (MatTek P35G-1.5-14-C). Three-dimensional (3D) d-STORM imaging was performed using Vutara SR352 microscope (Bruker) based on single-molecule localization biplane technology with lateral localization precision of 20–30 nm and axial precision of 60–70 nm. Imaging was conducted in the presence of an imaging buffer (7 μM glucose oxidase (Sigma), 56 nM catalase (Sigma), 2 mM cysteamine (Sigma), 50 mM Tris, and 10 mM NaCl, 10% glucose, pH 8). Images were recorded using 1.3 numerical aperture (NA) 60× silicon oil immersion objective (Olympus) and Hamamatsu Orca Flash 4v2 camera with frame rate at 50 Hz using 640-nm excitation laser (maximal excitation of 6 kW/cm2) with a collection of 9,000 frames. Data were analyzed by the Vutara SRX (version7.0.00rc24) software.

### BcR cluster analysis

BcRs displayed on the cell surface were analyzed using the Cluster Analysis Statistical Module of Vutara SRX software (version 7.0.00rc24). Cluster Analysis Module uses a DBSCAN cluster identification algorithm (22) designed to generate grouped clusters of localizations within the context of the larger data set. This algorithm directly uses particle distances and densities of the localization super resolution data to assign clusters and calculate their radius of gyration (the radial distance to the center of mass of a cluster). Cluster regions are grown from seed points to allow arbitrary shapes in the 3D data set. Cluster analysis was done by selecting the signal from the membrane area of each cell (approximately 500 nm in thickness) with the following parameter set: maximum particle distance of 100 nm (the threshold distance to determine if a particle is near enough to the other particle in a cluster to be a part of a cluster) and a minimum of 10 particle counts per cluster (the particle density, a cluster is only formed if the number of particles within the maximum particle distance of each other is greater than the minimum particle count). A total number of 24, 16, and 20 cells for IgG M-CLL, IgM M-CLL, and IgM U-CLL groups were analyzed, respectively.

### RNA sequencing

RNA was extracted from CD19^+^ CLL cells (IgG M-CLL, IgM M-CLL, and IgM U-CLL) using the RNeasy Mini Kit (Qiagen, Hilden, Germany) and RNA Clean & Concentrator kit (Zymo Research, CA, USA) according to the manufacturer’s instructions. Sequencing libraries were prepared using INCPM mRNA. Seventy-five–base pair (bp) reads were sequenced on one lane of NextSeq. The output was ~24 million reads per sample.

Bioinformatics: Poly-A/T stretches and Illumina adapters were trimmed from the reads using cutadapt (23); resulting reads shorter than 30 bp were discarded. Reads were mapped to the *H. sapiens* reference genome GRCh38 using STAR (24), supplied with gene annotations downloaded from Ensembl (and with EndToEnd option and outFilterMismatchNoverLmax was set to 0.04). Reads with the same UMI were removed using the PICARD MarkDuplicate tool using the BARCODE_TAG parameter. Expression levels for each gene were quantified using htseq-count (25), using the gtf above. Differentially expressed genes were identified using DESeq2 (26) with the betaPrior, cooksCutoff, and independentFiltering parameters set to False. Raw P-values were adjusted for multiple testing using the procedure of Benjamini and Hochberg. Pipeline was run using snakemake (27). The data for this study have been deposited in the European Nucleotide Archive (ENA) at EMBL-EBI under accession number PRJEB53802 (https://www.ebi.ac.uk/ena/browser/view/PRJEB53802).

### Statistical analysis

To compare between the two paired groups, within each type of experiment, the Student’s t-test was applied to compare the means of normal distributed dependent variables, and the Wilcoxon signed-rank test was applied to compare the distribution of non-parametric dependent variables. Mann-Whitney test was used to compare the distribution of unpaired non-parametric variables. All statistical analyses were performed using GraphPad Prism 9.0 software (GraphPad Software, San Diego, CA, USA). A P-value of <0.05 was considered as statistically significant.

## Results

### Diverse expression patterns of proximal BcR signaling proteins in distinct CLL immunogenetic subtypes

**Figure 1:**
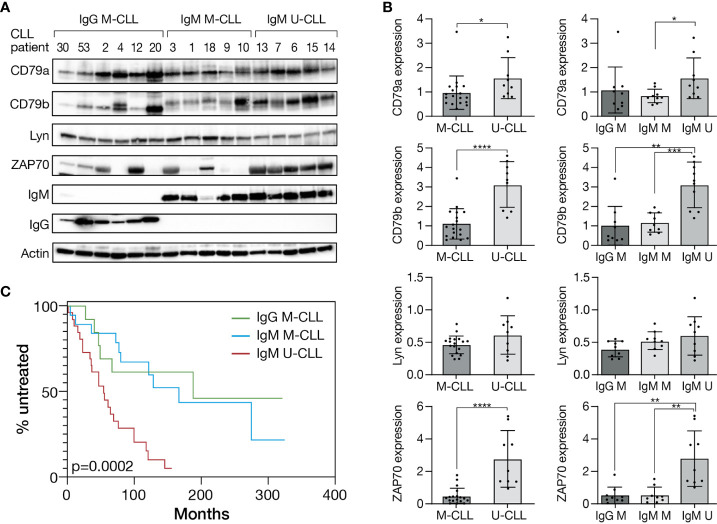
Different expression pattern of proximal BcR signaling proteins in CLL immunogenetic subtypes. **(A)** A representative Western blot analysis of primary CD19^+^ purified cells from M-CLL and U-CLL cases showing CD79a, CD79b, Lyn, ZAP70, IgM, and IgG levels. Actin was used to verify equal loading. **(B)** Quantification of CD79a, CD79b, Lyn, and ZAP70 levels in IgG M-CLL, IgM M-CLL, and IgM U-CLL cells in **(A)** by normalization to actin using myImageAnalysis™ Software (n = 27). *p < 0.05, **p < 0.01, ***p < 0.001, and ****p < 0.0001. **(C)** Kaplan–Meier analysis of time to first treatment according to IGHV gene SHM status and IG subclass(n = 58).

To explore differences in the expression level of proximal BcR pathway elements between IgG M-CLL, IgM M-CLL, and IgM U-CLL, proteins were extracted from CD19^+^ selected CLL cells and subjected to Western blot analysis. The expression levels of CD79a, CD79b, and ZAP70 were higher in U-CLL versus M-CLL (IgM or IgG), whereas no statistically significant difference was evident in the levels of Lyn among the groups (**Figures 1A, B**). Similar differences were observed in the sub-analysis comparing IgM U-CLL versus IgM M-CLL, and IgM U-CLL versus IgG M-CLL subtypes, except for CD79a levels that did not differ significantly between IgM U-CLL versus IgG M-CLL (**Figures 1A, B**). Moreover, total IgM levels were higher in U-CLL versus M-CLL (**Supplementary Figure 1**). There were no differences in CD79a, CD79b, ZAP-70, and Lyn levels between IgG versus IgM M-CLL. The median time to first treatment was shorter in patients with IgM U-CLL (54.03 months) compared with patients with IgM M-CLL (166.73 months) or IgG M-CLL (187.63 months), p = 0.0002 (**Figure 1C**).

### IgM U-CLL shows more robust constitutive BcR signaling compared with IgM and IgG M-CLL

**Figure 2:**
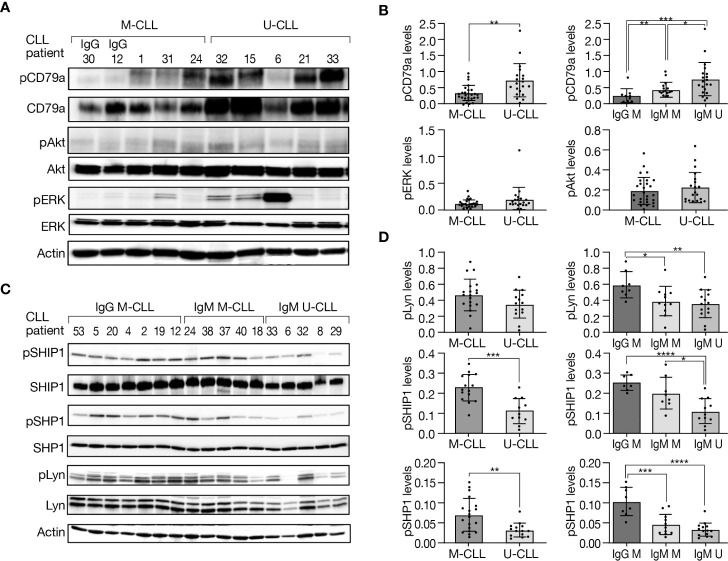
Basal phosphorylation levels of BcR signaling elements in M-CLL and U-CLL cells. **(A)** Representative Western blot analysis showing CD79a (Y182), Akt (S473), and ERK (T202/Y204) phosphorylation as well as total amount of these proteins. Actin was used to verify equal loading. **(B)** Quantification of pCD79a, pAkt, and pERK levels in **(A)** by normalization to actin using the myImageAnalysis™ Software (n = 47). *p < 0.05, **p < 0.01, and ***p < 0.001. **(C)** A Western blot analysis showing SHIP1 (Y1020), SHP1 (Y564), and Lyn (Y396) phosphorylation, as well as total amount of these proteins. Actin was used to verify equal loading. **(D)** Quantification of pSHIP1 (n = 26), pSHP1 and pLyn (n = 33) in **(C)** by normalization to actin using the myImageAnalysis™ Software. *p < 0.05, **p < 0.01, ***p < 0.001, and ****p < 0.0001.

To investigate differences in basal phosphorylation of BcR signaling elements between the IGHV subtypes and immunoglobulin subclasses, proteins were extracted from resting IgG M-CLL, IgM M-CLL, and IgM U-CLL, followed by Western blot analysis. CD79a was more intensely phosphorylated in U-CLL compared with M-CLL, whereas its phosphorylation levels were lowest in IgG M-CLL cases, higher in IgM M-CLL cases, and highest in IgM U-CLL (**Figures 2A, B**). Although no statistically significant difference was observed in autophosphorylation of Lyn at Y396 in the catalytic domain that is required for full catalytic activity of this kinase between M-CLL versus U-CLL, higher levels were detected in IgG M-CLL compared with IgM U-CLL or IgM M-CLL (**Figures 2C, D**). In addition, no statistically significant differences in phosphorylation of the inhibitory tyrosine (Y507) of Lyn were found between the BcR subgroups (**Supplementary Figures 2B, C**). BTK was more phosphorylated in U-CLL than in M-CLL and especially more in IgM U-CLL compared with IgG M-CLL cases (**Supplementary Figures 2A, C**). Moreover, no statistically significant differences were found in the basal phosphorylation levels of ERK and Akt between the three immunoglobulin subtypes/subclasses (**Figure 2B**). The basal phosphorylation of SHP1 and SHIP1, which are negative regulators of BcR signaling, was higher in M-CLL than U-CLL, a difference mostly driven by an increased phosphorylation in IgG M-CLL (**Figures 2C, D**). To this end, to exclude changes in phosphorylation during cell culture, we studied the baseline phosphorylation levels of CD79a, Akt, ERK, SHP1, and Lyn immediately after thawing and after a short-term incubation at 37°C. We found no statistically significant differences in phosphorylation levels immediately after thawing compared with their levels after 24 h of incubation at 37°C (**Supplementary Figure 3**). These findings confirm that a 24-h culture does not affect the basal phosphorylation levels of these proteins and can be considered in line with constitutive cell-intrinsic BcR signaling in CLL cells that is antigen-independent and more robust in IgM U-CLL.

### BcR signaling competence in immunogenetic subtypes of CLL

It is well recognized that U-CLL is typically BcR competent, whereas M-CLL is often anergic. In an attempt to obtain more evidence into this issue and also explore potential differences depending on the IG subclass, we evaluated the response to BcR engagement in IgG M-CLL, IgM M-CLL, and IgM U-CLL. CLL cells were incubated with either goat F(ab′)2 anti-human IgM or F(ab′)2 anti-human IgG for 15 min or left untreated. This activation time point was selected after we performed time-dependent BcR activation experiments, in which we observed that 15 min is the maximal activation point (**Supplementary Figure 4**).

**Figure 3:**
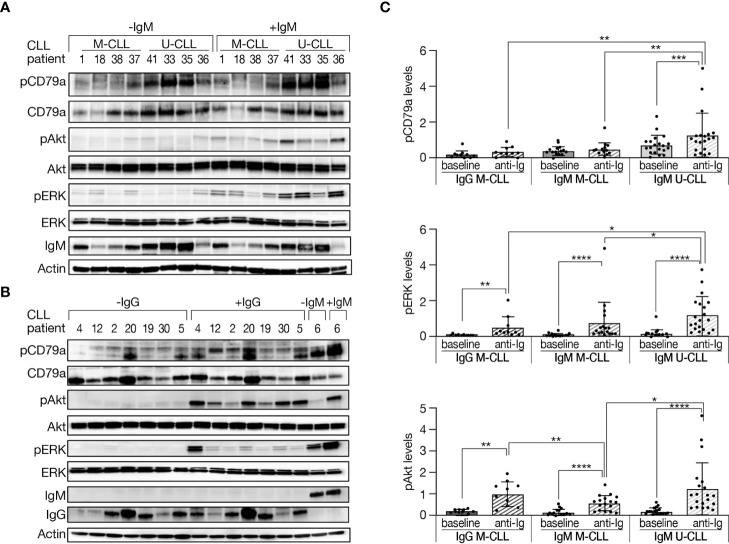
Phosphorylation levels of BcR signaling elements in M-CLL and U-CLL cells after BcR activation. **(A, B)** Peripheral blood CLL cells were incubated with goat F(ab′)2 anti-human IgM or with goat F(ab′)2 anti-human IgG (10 µg/ml) for 15 min or left untreated. Protein was extracted and analyzed by Western blot. A representative Western blot analysis showing CD79a (Y182), Akt (S473), and ERK (T202/Y204) phosphorylation, as well as total amount of these proteins, and IgM and IgG levels. Actin was used to verify equal loading. **(C)** Quantification of pCD79a, pAkt, and pERK levels in activated and inactivated CLL cells in **(A, B)** by normalization to actin using the myImageAnalysis™ Software. (n = 47).*p < 0.05, **p < 0.01, ***p < 0.001, and ****p < 0.0001.

Phosphorylation levels of CD79a, ERK, and Akt were measured in cells at baseline and after activation. As shown in **Figures 3A, C**, the increase in proteins phosphorylation, especially CD79a, after BcR activation was more robust in IgM U-CLL compared with IgM M-CLL. Likewise, the phosphorylation levels of CD79a and ERK following BcR activation were higher in IgM U-CLL compared with IgG M-CLL subgroup (**Figures 3B, C**). No statistically significant differences were observed in CD79a and ERK phosphorylation after BcR activation in IgG versus IgM M-CLL. The phosphorylation levels of Akt after BcR activation were not statistically different between IgG M-CLL and IgM U-CLL but were both stronger than in IgM M-CLL (**Figure 3C**). Overall, these findings suggest that both IgG and IgM M-CLL are less responsive to BcR cross-linking compared with IgM U-CLL.

### The gene expression signature of IgG class-switched M-CLL is closely related to IgM M-CLL

**Figure 4:**
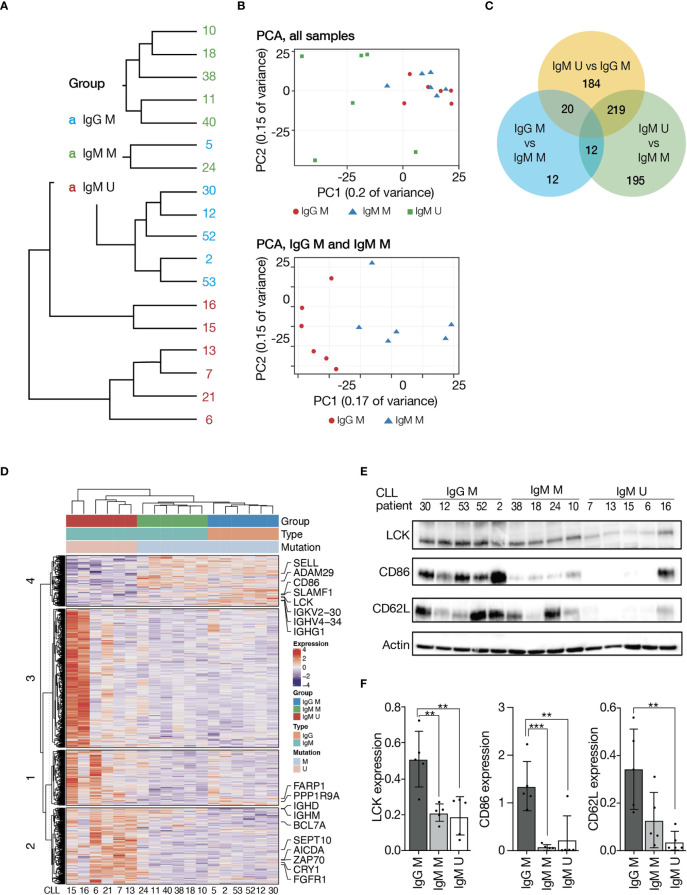
Gene expression profiling of CLL immunogenetic subgroups. RNA was extracted from unstimulated CD19^+^ CLL cells. Sequencing libraries were prepared using INCPM mRNA. **(A)** Unsupervised hierarchical clustering. **(B)** Principal component analysis. **(C)** Venn diagram showing the number of genes differentially expressed between the three subgroups. **(D)** Heatmap of genes differentially expressed between the three subgroups (>2-fold change, padj ≤ 0.05). Gene expression is median centered and scaled as indicated. **(E)** Representative Western blot analysis of IgM or IgG M-CLL and IgM U-CLL cells showing LCK, CD86, and CD62L levels. Actin was used to verify equal loading. **(F)** Quantification of LCK, CD86, and CD62L levels in IgG M-CLL, IgM M-CLL, and IgM U-CLL cells in **(E)** by normalization to actin using the myImageAnalysis™Software (n = 15). **p < 0.01 and ***p < 0.001.

To characterize the gene expression of IgG class-switched versus IgM^+^ CLL, we performed RNA sequencing (RNAseq) analysis of 18 CD19^+^ isolated CLL cell samples on Illumina sequencing platform. All IgG^+^ CLL samples in this analysis carried mutated IGHV4-34 gene rearrangements. After normalization, unsupervised clustering clearly separated the samples primarily according to IGHV gene SHM status and then by the IG subclass (**Figures 4A, B**). Next, we directly compared (supervised analysis) the three different CLL subtypes/subclasses (IgM U-CLL, IgM M-CLL, and IgG M-CLL). CLL cells differentially expressed 642 genes, whose expression levels were at least two-fold different between IgM U-CLL, IgM M-CLL, and IgG M-CLL. Of these, 353 genes were upregulated in IgM U-CLL and 70 were downregulated compared with IgG M-CLL. Using the same criteria, only 44 genes were differentially expressed between IgG and IgM M-CLL, 20 being more highly expressed in IgM M-CLL and 24 showing reduced expression (**Figures 4C, D**). We further validated the RNAseq results by Western blot analysis, showing overexpression of LCK, CD86, and SELL (CD62L) in IgG M-CLL (**Figures 4E, F**). To investigate the biologic basis of these gene expression differences, we used Gene Set Enrichment Analysis (GSEA). This observer-independent statistical method can dissect gene expression data into common functional pathways. Using this method, we identified activation of distinct signaling pathways in IgM U-CLL compared with IgM M-CLL and IgG M-CLL, including BcR signaling, NF-κB signaling, MAPK-ERK signaling, and others (**Supplementary Tables 2–4**). Of the most enriched pathways in the U-CLL group, the activation of the BcR pathway is supported by this work results and the literature. On the other hand, no difference was found in the baseline phosphorylation levels of p65 between the three immunoglobulin subtypes/subclasses (**Supplementary Figures 2A, C**).

### Distinct BcR localization and dynamics in CLL immunogenetic subtypes

To determine differences in the localization and dynamics of the BcR and the actin cytoskeleton in resting CLL cells and after activation, CLL cells were seeded on poly-L-lysine–coated glass, then left either inactivated or activated with F(ab′)2 Fragment anti-IgM or anti-IgG for 5, 15, and 40 min. The cells were then fixed, permeabilized, and labeled with FITC-conjugated anti-IgM or anti-IgG and DyLight 594 Phalloidin for F-actin. Images were acquired using confocal microscope.

**Figure 5:**
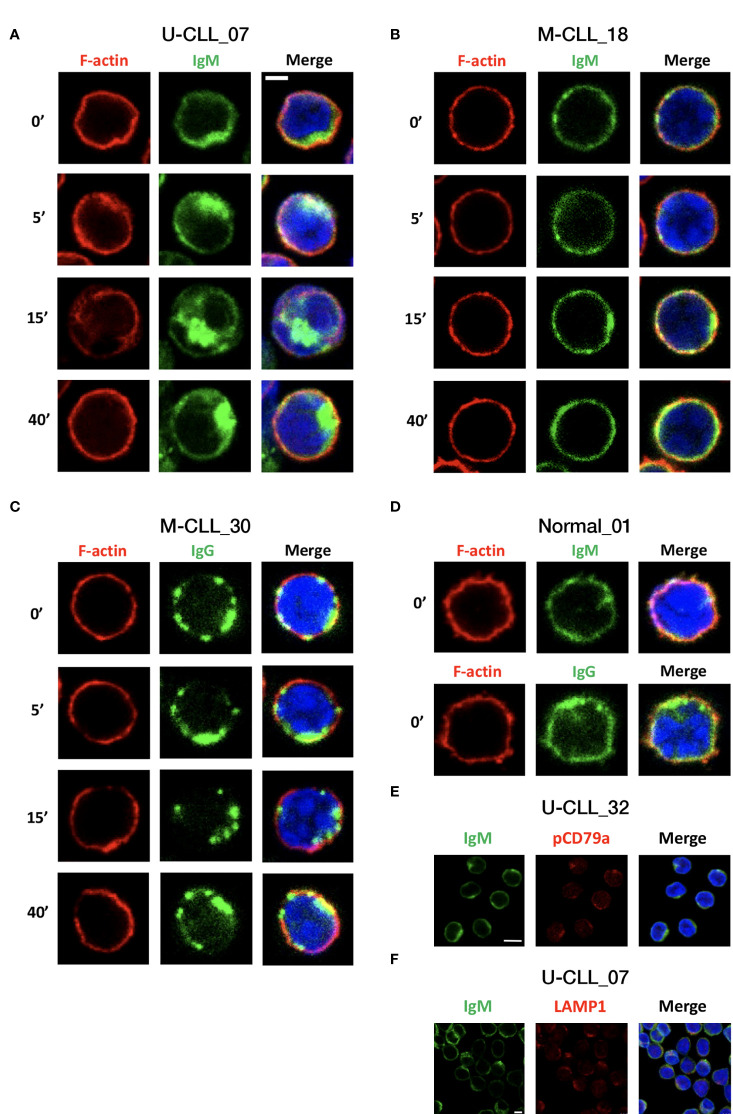
IgM, IgG, and F-actin distribution and localization before and after activation. CLL cells were seeded on poly-L-lysine–coated glass and then left either inactivated or activated with F(ab′)2 Fragment anti-IgM or F(ab′)2 Fragment anti-IgG for 5, 15, and 40 min. The cells were fixed and permeabilized, followed by staining. The images presented are confocal slices through the center of the cell. **(A)** Representative IgM U-CLL case. **(B)** Representative IgM M-CLL case. **(C)** Representative IgG M-CLL case. **(D)** Representative case of IgM and IgG staining in normal B cells. Scale bar, 2 μm. **(E)** Unstimulated CLL cells were stained for IgM and pCD79a. Representative images of patient with U-CLL are shown. Scale bar, 5 μm (n = 3). **(F)** Unstimulated CLL cells were stained for IgM and LAMP1. Representative images of patient with U-CLL are shown. Scale bar, 2.5 μm (n = 4).

The results of 25 cases demonstrate substantial differences between samples. In resting IgM M-CLL, the IgM was organized diffusely across the cell membrane, whereas in IgM U-CLL, it was commonly clustered into polarized patches and even in caps, and, occasionally, the BcRs were observed inside the cells (**Figures 5A, B**). When comparing the BcR organization in IgM versus IgG M-CLL, IgG appeared more clustered throughout the cell membrane, but no internalization was observed (**Figures 5B, C**). In resting B cells of healthy donors, both IgM and IgG were diffusely organized along the cell membrane in a similar pattern to that observed in IgM M-CLL (**Figure 5D**). After BcR activation, most IgM U-CLL samples showed prominent IgM reorganization into large clusters forming polarized caps, internalization and, later on, a return of the IgM to the cell surface after 40 min (**Figure 5A**). In contrast, most IgM and IgG M-CLL samples did not show significant dynamics of the BcR spatial distribution following activation (**Figures 5B, C**).

To explore F-actin cytoskeleton dynamics, we analyzed phalloidin intensity and co-localization with IgM/IgG at 5, 15, and 40 min after BcR activation. In U-CLL, the phalloidin intensity and co-localization with BcR showed a tendency to increase at 5 min after activation, decrease at 15 min, and return to baseline levels at 40 min; however, these differences did not reach statistical significance. Overall, no clear changes in F-actin organization were observed in M-CLL. In accordance with the actin cytoskeleton dynamics, at 15 min after BcR activation, the cell area slightly increased only in U-CLL (**Supplementary Figure 5A**). In addition, we counted the cells with BcR’s capping or internalization in the basal state and showed higher percentages in the IgM U-CLL compared with IgM/IgG M-CLL groups (**Supplementary Figure 5B**). We also show co-localization of IgM and pCD79a in resting CLL cells (**Figure 5E**). To confirm BcR internalization, we co-stained the cells with anti-IgM and LAMP1, a lysosomal membrane glycoprotein. Our results indicate that there is a co-localization between IgM and the lysosome marker (**Figure 5F**).

**Figure 6:**
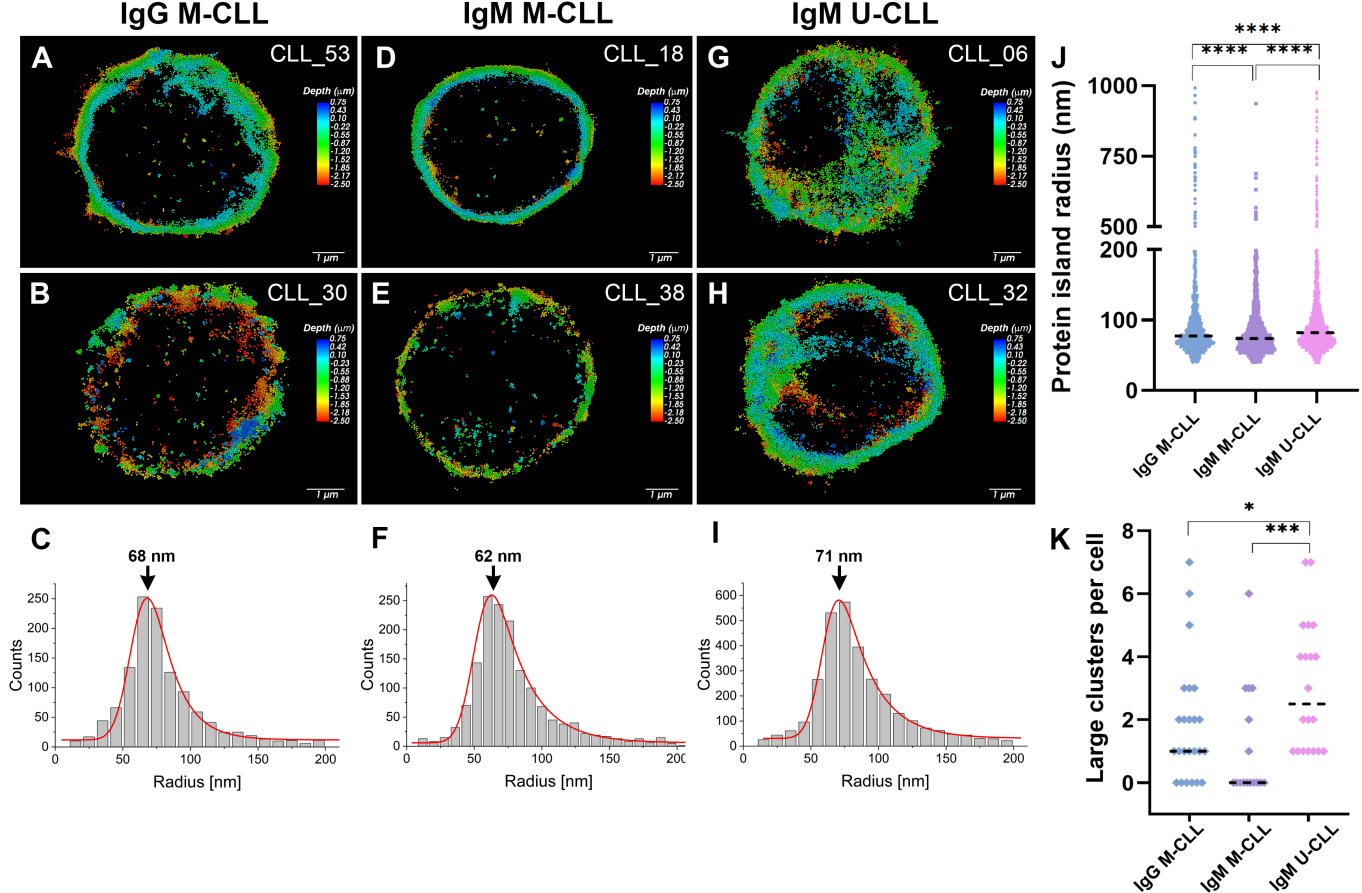
IgM and IgG imaging and cluster analysis using d-STORM. Typical three-dimensional d-STORM images of **(A, B)** patients with IgG M-CLL, **(D, E)** patients with IgM M-CLL, and **(G–H)** patients with IgM U-CLL. Images are x-y projections of individual BcR localizations, in which the depth (distance along the z axis) is shown in color coded manner. **(C, F, I)** Statistical distribution of BcR small cluster size (range of 40–200 in radius) in IgG M-CLL, IgM M-CLL, and IgM U-CLL groups, respectively. The peak value of the Gaussian fit (in red) is marked with an error. **(J)** Radius distribution of protein islands at the cell membrane for IgG M-CLL, IgM M-CLL, and IgM U-CLL; median is marked in a black dashed line. ****p < 0.0001. **(K)** Number of large clusters (500–1,000 nm) per cell for IgG M-CLL, IgM M-CLL, and IgM U-CLL; mean value with one standard deviation is marked in black. *p < 0.05 and ***p < 0.001.

Examination of BcR organization and distribution in resting CLL cells was also performed by direct stochastic optical reconstruction microscopy (d-STORM) that reconstructs super resolution fluorescence images in three dimensions with a lateral resolution of the order of 20–30 nm and axial resolution of 60–70 nm. **Figure 6** shows representative super resolution images of IgG M-CLL, IgM M-CLL, and IgM U-CLL subgroups. Whereas IgG M-CLL (**Figures 6A, B**) and IgM M-CLL (**Figures 6D, E**) display labeling restricted to the membrane plane of the cells, in IgM U-CLL, labeling is also observed within the cells, as indicated by the signal located in the central part of the cells (**Figures 6G, H**). Representative 3D reconstructions demonstrate the differences of the basal BcR distribution between IgM U-CLL (**Supplementary Movie S1**), IgM M-CLL (**Supplementary Movie S2**), and IgG M-CLL (**Supplementary Movie S3**).

Quantitative analysis of BcR organization was performed by molecular cluster analysis along the membrane of the cells. The median radius of BcR protein islands (40–1,000 nm) on resting CLL cells was larger in IgM U-CLL than in IgM and IgG M-CLL as well as larger in IgG M-CLL compared with IgM M-CLL (**Figure 6J**). The BcR clusters had a wide range of sizes, with a distribution that is strongly skewed toward smaller cluster size (smaller than 200 nm) for all three subgroups (**Figures 6C, F, I**). The mean values for the radii of these small clusters, which make up about 90% of all the clusters on all CLL subtypes, do not differ significantly between the cell types. The main differences between the CLL subtypes are the number and sizes of the large clusters with larger cluster radii. Whereas IgM M-CLL had the narrowest size distribution, the cluster distributions of IgM U-CLL and IgG M-CLL are wider and contain higher number of large clusters with radius of 500–1,000 nm. **Figure 6K** represents the number of large clusters per cell for each sample. IgM U-CLL cells have the highest tendency for large clusters, whereas IgM M-CLL have the lowest tendency for large clusters. An example of both large and small clusters for IgM U-CLL cell is shown in **Supplementary Figure 6**.

## Discussion

In this study, we demonstrate distinct differences in the early BcR signalosome, signaling responses, and spatial organization between CLL immunogenetic subtypes (**Supplementary Figure 7**). In particular, we show that the IgG class-switched M-CLL is biologically closely related to IgM M-CLL and has a similar favorable clinical outcome.

In B cells, CD79a forms a heterodimer with CD79b, associated with membrane-bound IG, which is required for BcR signaling (28–31). Here, we show a more robust basal phosphorylation of CD79a in U-CLL versus M-CLL. Phosphorylation levels of CD79a were lowest in IgG M-CLL, higher in IgM M-CLL, and highest in IgM U-CLL. Given that CD79a is a key component of the BcR, our findings are indicative of constitutive cell-intrinsic BcR signaling in CLL, which is more robust in the more clinically aggressive U-CLL subtype. These findings are consistent with the cell-autonomous BcR signaling model in CLL, derived through inter-molecular interactions between the distinct IG molecules (14). We further show increased phosphorylation of SHP1 and SHIP1, negative regulators of the BcR pathway, in resting M-CLL, in particular in the IgG subclass. These results are consistent with a previous report showing that continuous activities of both SHP1 and SHIP1 are required to maintain anergy, which is mediated through independent pathways (32). Accordingly, SHIP1 negatively regulates the PI3K/Akt signaling, whereas SHP1 dephosphorylates several key proteins in the BcR pathway, including CD79a/CD79b, Syk, and BLNK (32–34). In addition, we show increased autophosphorylation of Lyn at Y396 in the catalytic domain that is required for full catalytic activity of this kinase in IgG M-CLL compared with IgM U-CLL or IgM M-CLL, but with no differences in phosphorylation of the inhibitory tyrosine (Y507). Given that Lyn is a double-edged kinase with a positive and negative regulator for antigen receptor-mediated signals, our results highlight the complexity of interpreting the impact of Lyn on the BcR signaling (35, 36).

BcR clustering is crucial for the activation of the BcR signaling pathway (37). We show that in resting U-CLL, IgM is frequently clustered in the membrane plane within polarized patches, occasionally in caps, and sometimes even inside the cells. Within M-CLL, IgM is scattered diffusely along the membrane plane in a similar pattern to normal B cells, whereas IgG is dispersed around the cell membrane in smaller clusters than in IgM U-CLL cells (**Supplementary Figure 7**). Furthermore, we show that IgM clusters in unstimulated cells co-localize with pCD79a, which further reflect activated BcR complex aggregation. Altogether, the results of our experiments indicate that increased BcR clustering is associated with increased BcR signaling.

By using d-STORM microscopy, a super-resolution technique that enables mapping nanoscale spatial organization at the level of individual BcRs, we show that the basal radius of the BcR protein islands in IgM U-CLL is larger than in IgM or IgG M-CLL. It has been previously described in normal B cells that, after antigen engagement, the radii of both IgM BcR and IgG BcR islands increase (38). The change in the protein islands radius size expresses the formation and organization of the signalosome that is important for the activation of the BcR and the subsequent activation of downstream signaling. Thus, the increased protein islands radius in the IgM U-CLL group indicates high basal activity.

We further confirmed by d-STORM microscopy that IgM is localized occasionally inside the cells in U-CLL. Although it has been previously reported that resting CLL cells are more likely to be organized in microclusters than normal B cells (39), our findings reveal distinct patterns according to the IGHV gene SHM status and immunoglobulin subclass usage. By using d-STORM imaging, it has been reported that IgG BcRs are more clustered than IgM BcRs on resting normal B cells and form larger protein islands after antigen activation (38). Altogether, the BcR spatial organization pattern and the biochemical data presented in this study point to high basal activity of the BcR in the more aggressive group of patients with U-CLL in the more aggressive group of U-CLL patients.

CLL cells typically express lower surface levels of CD79b (40, 41) and, conversely, higher levels of Lyn (42) than normal B cells. Total CD79a expression was lower in both IgM and IgG M-CLL, but a statistically significantly difference in total CD79a levels was observed only between IgM M-CLL versus IgM U-CLL. Lyn levels did not differ between the three subgroups. High CD79b expression has been associated with strong surface IG expression, advanced clinical stage, and short overall survival in CLL (40, 43). Similar to our results, Guo et al. (41) have shown that M-CLL samples express less total CD79b protein than U-CLL samples. In addition, Guo et al. (41) reported that CLL cells express relatively normal levels of total CD79a and IgM and proposed that deficient CD79b expression leads to decoupling with CD79a and IgM, which blocks BcR assembly and reduces surface IgM expression in CLL cells (41). The latter may be consistent with lower surface IgM and IgG expression in M-CLL compared with U-CLL (44). Moreover, CLL cells have been shown to exhibit glycosylation and folding defects of the IgM and CD79a, which may further impair BcR assembly and reduce surface IgM. Nevertheless, we show considerable lower total IgM expression levels in M-CLL versus U-CLL, which might be linked, at least to some extent, to the lower CD79a levels in the former. Of note, we found a notable heterogeneity among patients with IgG M-CLL, IgM M-CLL, and IgM U-CLL. This is in agreement with previous studies and reflects the different genetic/clinical background of individual patients. However, the differences between the CLL groups were significant and profound.

Furthermore, we show distinct gene expression signatures between the three immunogenetic subgroups. The gene expression signature of IgG M-CLL was closely related to that of IgM M-CLL, whereas both were distinct from IgM U-CLL. Thus, the gene expression of CLL cells seems to be primarily associated with the IGHV gene SHM status and only to a lesser extent with the IG subclass. Many of the genes found to be differentially expressed between IgM U-CLL and IgG M-CLL cells were previously reported to differ between U-CLL versus M-CLL (e.g., ZAP70, SEPT-10, BCL7A, AICDA, ADAM29, and CD86) (45, 46). Using a Western blot analysis, we showed overexpression of CD62L, CD86, and LCK in IgG M-CLL. CD62L is an adhesion molecule and plays an important role in the traffic and homing of lymphocytes to lymph nodes (47). It has already been shown that stimulation through BcR causes a decrease of CD62L, especially in patients with a poor prognosis and risk of disease progression (48). Our results showing higher expression of CD62L in the less aggressive group of CLL are consistent with this claim. CD86 is a molecule that interacts with the CD28 receptor on T cells for co-stimulatory signals. A previous study has shown that DNA methylation levels for CD86 is significantly higher in subset #1 than subset #4 CLL samples, in accordance with lower CD86 gene and protein expression in the more aggressive group of CLL (46). These findings are consistent with our results, as most patients with IgG M-CLL included in the analysis belong to subset #4. Another study has shown that LCK plays a major role in maintaining the anergic phenotype in CLL, consistent with its lower expression in U-CLL in our study (49). Among the genes differentially expressed in IgM U-CLL cells, GSEA identified several significantly enriched gene sets indicating activation of distinct signaling pathways compared with IgM and IgG M-CLL cells, particularly BcR, NF-κB, and MAPK-ERK signaling pathways. The NF-κB pathway is known to be constitutively activated in CLL cells. Cell-intrinsic events and external stimulation from the micro-environment through various receptors, for example, BcR, TLR, and CD40, are closely linked to downstream activation of NF-κB in CLL (50). Gene expression profiling of CLL cells from different compartments of peripheral blood, bone marrow, and lymph nodes revealed that the lymph node microenvironment promotes BcR signaling and NF-κB activation, eventually sustaining CLL proliferation and survival *in vivo* (5). Furthermore, higher baseline p65 (Rel A) activity is strongly associated with advanced clinical stage and disease progression in patients with CLL (51). Taken together, the above literature is consistent with our RNAseq results showing enrichment of the NF-κB signaling pathway in the more aggressive group of CLL. In the future, there is more work to be done to characterize the constitutively activated elements of the NF-kB pathway in CLL.

In addition to the increase in the basal activity of the pathway, BcR responsiveness is enhanced in U-CLL versus either IgM or IgG M-CLL. Whereas the degree of phosphorylation of CD79a, ERK, and Akt after BcR activation is reduced in IgM M-CLL compared with IgM U-CLL, only phosphorylation of CD79a and ERK were attenuated in IgG M-CLL. These results are consistent with previous studies showing higher BcR responsiveness of U-CLL to IgM ligation, versus a relatively “anergic” behavior of M-CLL cells (6, 52), that also correlates with ectopic expression of ZAP70 in the former (53). Interestingly, in IgG^+^ CLL, the increase in Akt and ERK phosphorylation after BcR activation was noted although CD79a phosphorylation levels did not change significantly. This result may possibly be attributed to the fact that IgG BcRs can initiate intracellular signals also through a conserved tyrosine residue in the cytoplasmic segments of IG heavy chains. When phosphorylated, this tyrosine recruits the adaptor Grb2, resulting in protein kinase activation and generation of second messengers (54, 55). In addition, IgM U-CLL cells showed prominent IgM reorganization into large clusters forming polarized caps and internalization after BcR activation, whereas no significant reorganization or internalization of the BcR was observed in IgM and IgG M-CLL cells following activation. These findings are consistent with previous reports that BcR-mediated signaling regulates the growth of BcR microclusters (56) and internalization (57–59).

Overall, the results of our study shed light on the biochemical status and dynamics of key elements in the BcR in the context of immunogenetic status and clinical behavior, with an emphasis on the IgG M-CLL group. We report fundamental differences in the basal composition, biochemical status, and spatial organization of the BcR in the three examined immunogenetic CLL subtypes that correlate with their clinical behavior. Although U-CLL shows a more robust constitutive BcR pathway activation and a better responsiveness to BcR engagement, our data suggest that the BcR pathway is also a therapeutic target in IgG CLL. Moreover, we provide evidence that class-switched M-CLL likely represents the same disease as IgM M-CLL rather than a different biological and/or clinical entity.

## Data availability statement

The datasets presented in this study can be found in online repositories. The names of the repository/repositories and accession number(s) can be found below: https://www.ebi.ac.uk/ena/, PRJEB53802.

## Ethics statement

The studies involving human participants were reviewed and approved by the Tel-Aviv Sourasky Medical’s Institutional Review Board. Cells were obtained from peripheral blood samples donated by patients fulfilling the standard criteria for CLL after signing informed consent according to the Helsinki Accords. The patients/participants provided their written informed consent to participate in this study.

## Author contributions

YSA performed the experiments, analyzed data, and wrote the paper. YB performed the experiments and analyzed data. TD performed d-STORM experiments, analyzed data, and wrote the paper. TK and MS performed the experiments. ST analyzed data. YH and B-ZK initiated the study, supervised the research, analyzed data, and wrote the paper. All authors contributed to the article and approved the submitted version.

## Acknowledgments

YH is supported by grants from the Israeli Science Foundation (1707/19), Israeli Cancer Association, and Sackler Faculty of Medicine, Tel-Aviv University. We thank Prof. Kostas Stamatopoulos for critical reading of the manuscript and constructive comments. We thank Dr. Eviatar Weizman from Weizmann Institute of Science for the RNAseq analysis. We also thank The Mantoux Bioinformatics institute and The Crown Genomics institute of the Nancy and Stephen Grand Israel National Center for Personalized Medicine, Weizmann Institute of Science. D-STORM microscopy was performed at the Irving and Cherna Moskowitz Center for Nano and Bio-Nano Imaging at the Weizmann Institute of Science.

## Conflict of interest

YH received honoraria from AstraZeneca, Janssen, AbbeVie, and Medison for work unrelated to the present study.

The remaining authors declare that the research was conducted in the absence of any commercial or financial relationships that could be construed as a potential conflict of interest.

## Publisher’s note

All claims expressed in this article are solely those of the authors and do not necessarily represent those of their affiliated organizations, or those of the publisher, the editors and the reviewers. Any product that may be evaluated in this article, or claim that may be made by its manufacturer, is not guaranteed or endorsed by the publisher.
