# Supplementary material for: Spatial organization and early signaling of the B-cell receptor in CLL
Source: Front Immunol. 2022 Aug 9;13:953660. doi: 10.3389/fimmu.2022.953660 (PMC9398492; doi:10.3389/fimmu.2022.953660)
Supplement: Supplementary file 1 [file DataSheet_1.pdf]

Supplementary Figures

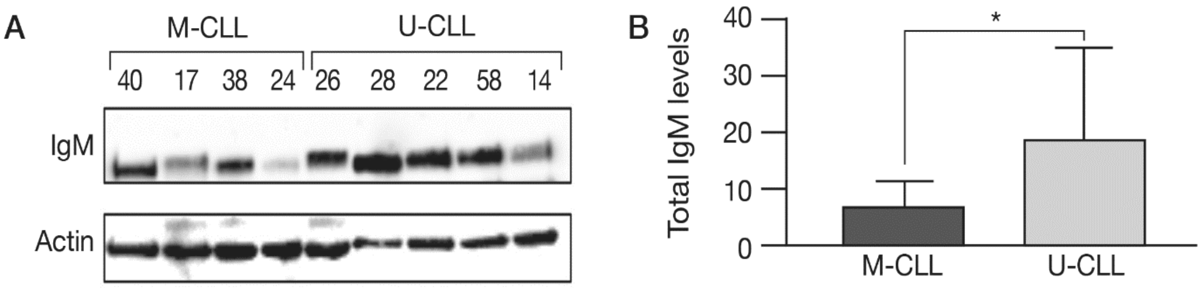

Supplementary Figure S1: IgM levels in M-CLL and U-CLL cells. A. A representative Western blot analysis of primary CD19+ purified cells from M-CLL and U-CLL cases showing IgM levels. Actin was used to verify equal loading. B. Quantification of IgM levels in M-CLL and U-CLL cells in (A) by normalization to actin using myImageAnalysis™ Software (n=23). *p<0.05.

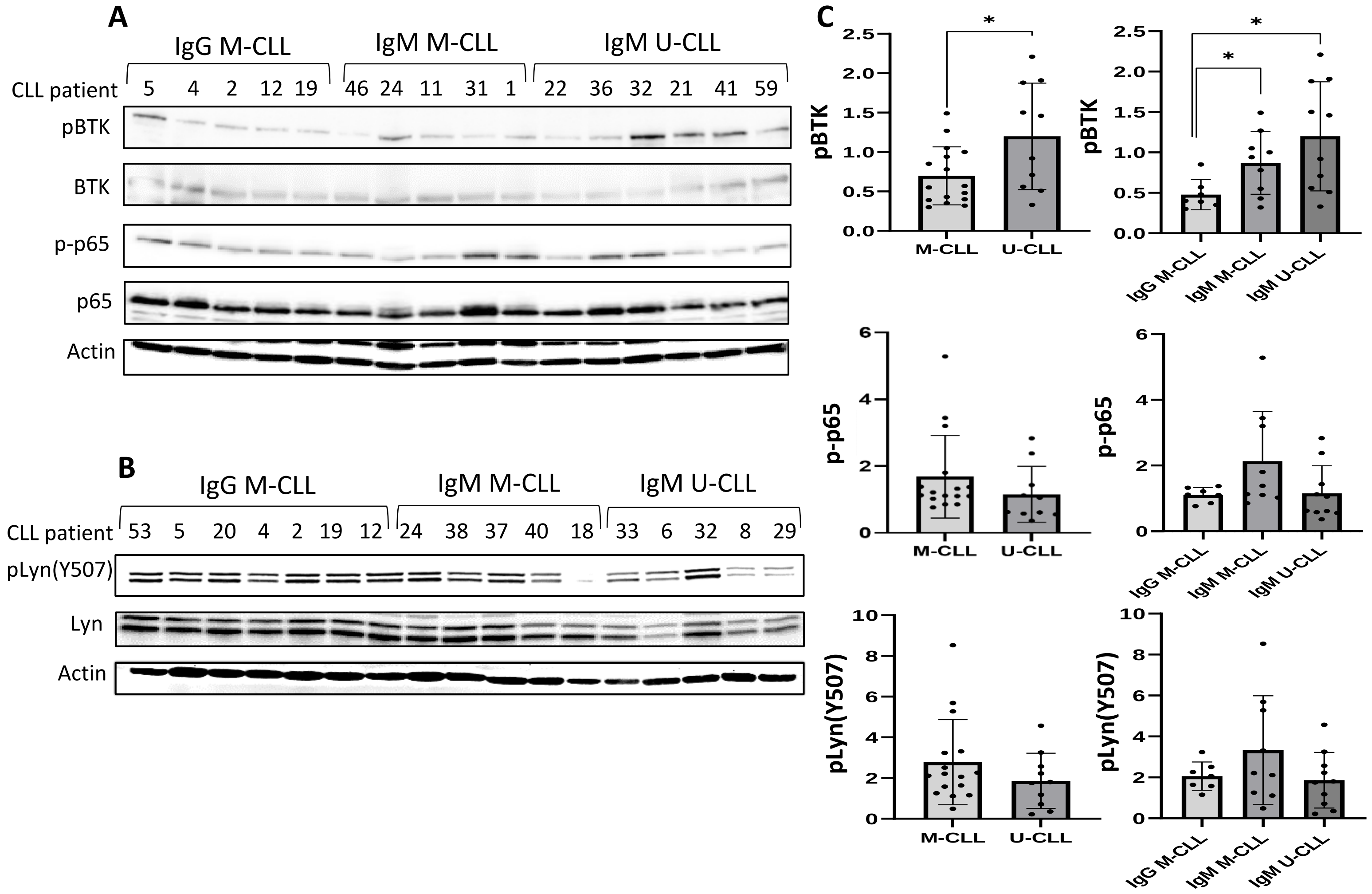

Supplementary Figure S2: BTK , p65 and Lyn phosphorylation in CLL subgroups. Peripheral blood CLL cells were isolated from IgG M-CLL, IgM M-CLL and IgM U-CLL patients. Protein was extracted and analyzed by Western blot.A. A representative Western blot analysis showing BTK(Y223) and p65(S536) phosphorylation. B. A representative Western blot analysis showing Lyn(Y507)

phosphorylation. Actin was used to verify equal loading. C. Quantification of pBTK, p-p65 and pLyn levels in (A) and (B) by normalization to actin using myImageAnalysis™ Software (n=26). *p<0.05.

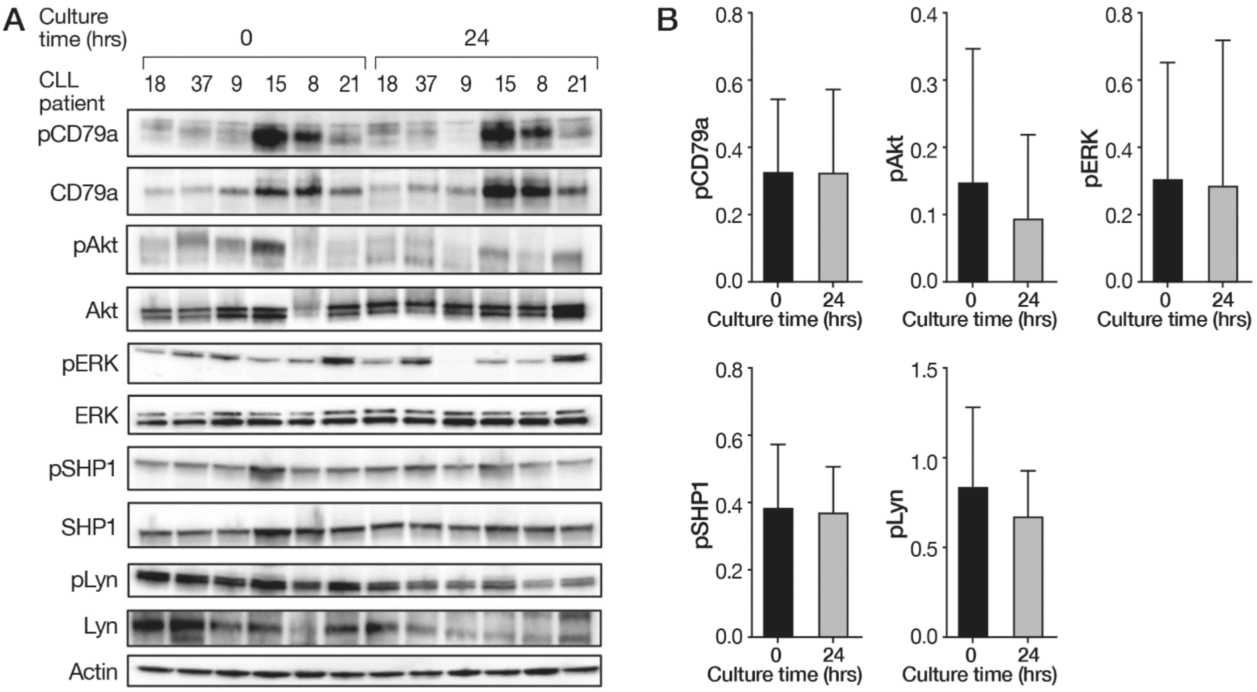

Supplementary Figure S3: Phosphorylation levels of BcR signaling elements in different time points. Peripheral blood CLL cells were isolated from M-CLL and U–CLL patients.Protein was extracted immediately after thawing and following 24 hours of incubation, and analyzed by Western blot. A. A representative Western blot analysis showing CD79a(Y182), Akt (S473), ERK(T202/Y204), SHP1 (Y564) and Lyn (Y396) phosphorylation, as well as total amount of these proteins. Actin was used to verify equal loading. B. Quantification of pCD79a, pAkt, pERK, pSHP1 and pLyn levels in (A) by normalization to actin using myImageAnalysis™ Software (n=14).

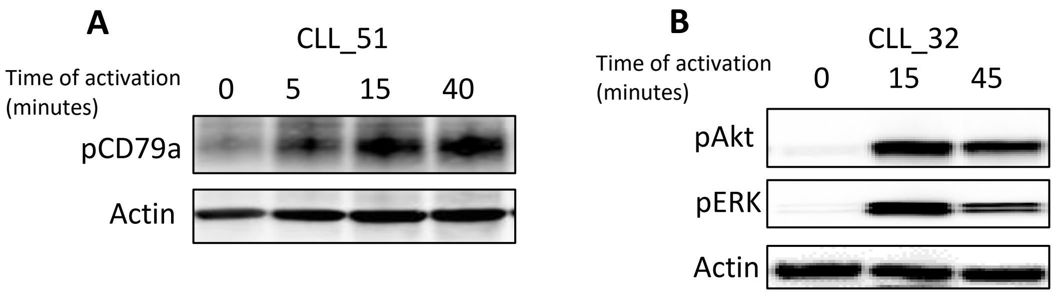

Supplementary Figure S4 : A time-dependent BcR activation pattern in CLL cells. CLL cells were incubated with goat F(ab')2 anti-human IgM (10 µg/mL), for the indicated time points or left untreated. Protein was extracted and analyzed by Western blot.A. A representative Western blot analysis showing CD79a(Y182) phosphorylation after 5, 15 and 40 minutes. Actin was used to verify equal loading (n=3). B. A

representative Western blot analysis showing Akt (S473) and ERK (T202/Y204) phosphorylation after 15 and 45 minutes. Actin was used to verify equal loading (n=8).

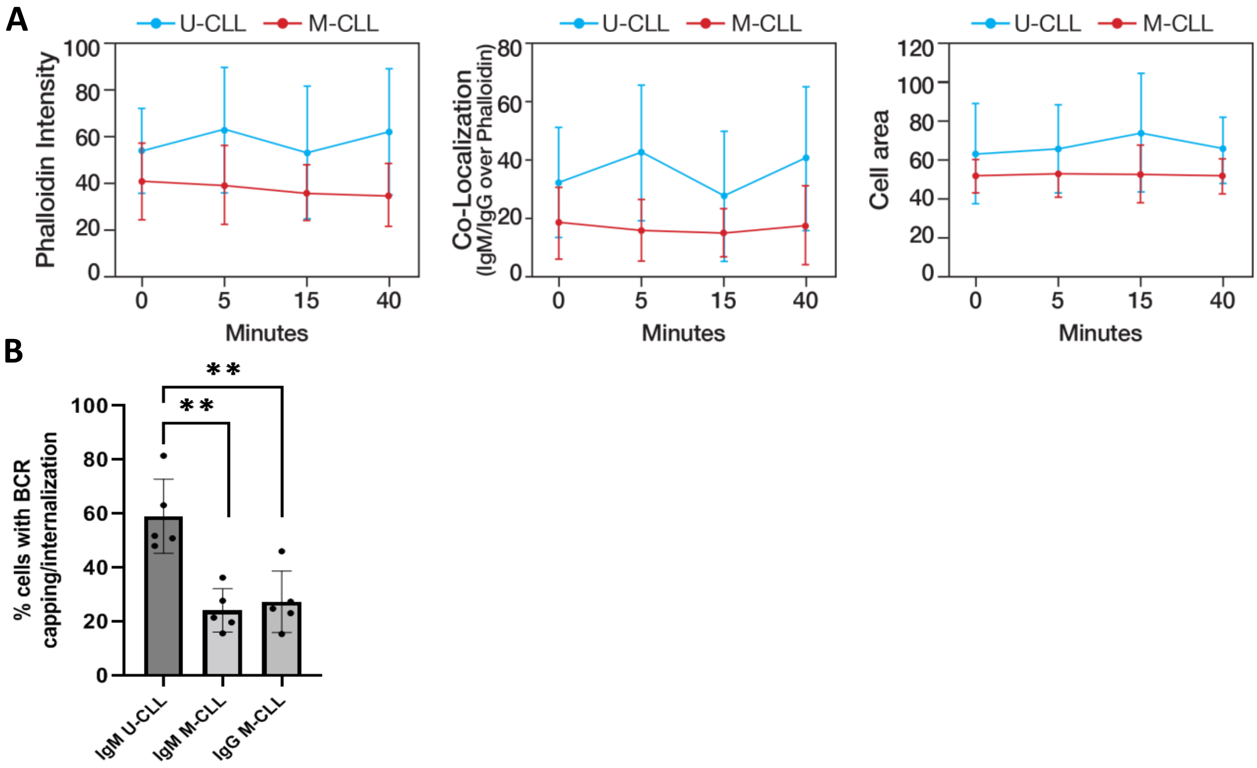

Supplementary Figure S5: BcRs and F-actin dynamic in M-CLL and U-CLL cells before and after activation. A.CLL cells were unstimulated ("0") or activated with F(ab')2 Fragment Goat Anti-Human IgM or IgG at 10µg/mL for 5, 15 and 40 minutes. The cells were stained with human anti-IgM or anti-IgG antibody conjugated to FITC, followed by staining with DyLight 594 Phalloidin. Quantification of Phalloidin intensity,Co-Localization of IgM/IgG over Phalloidin and cell area was done using Imaris software (n=25). B. Analysis of the percentage of cells in the basal state showing capping/ internalization of the BcR's in the three groups of CLL (n=15, mean of two fields per patient) **p<0.01.

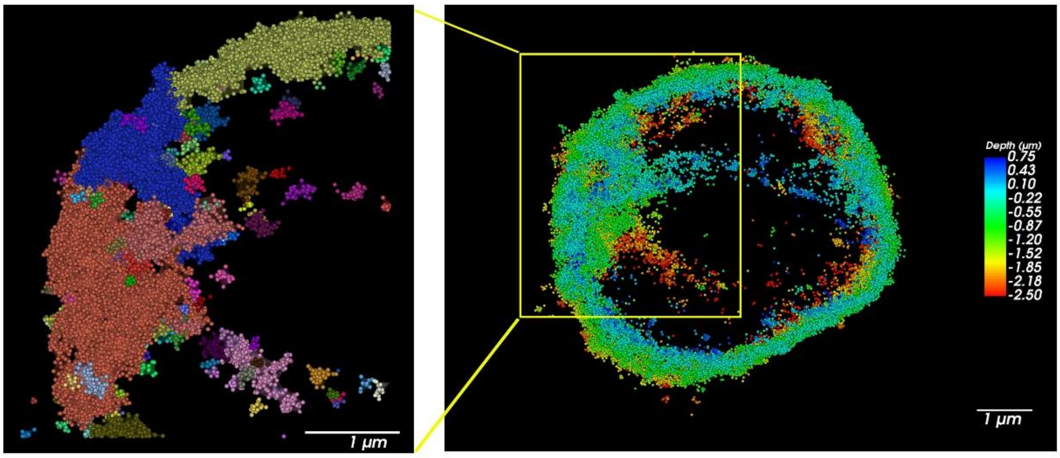

Supplementary Figure S6: Visualization of cluster analysis of d-STORM data. Depth representation of a d-STORM image of IgM U-CLL cell (right panel) and its corresponding clusters (left panel). This cell contains both large and small clusters, each individual cluster is marked in a different color.

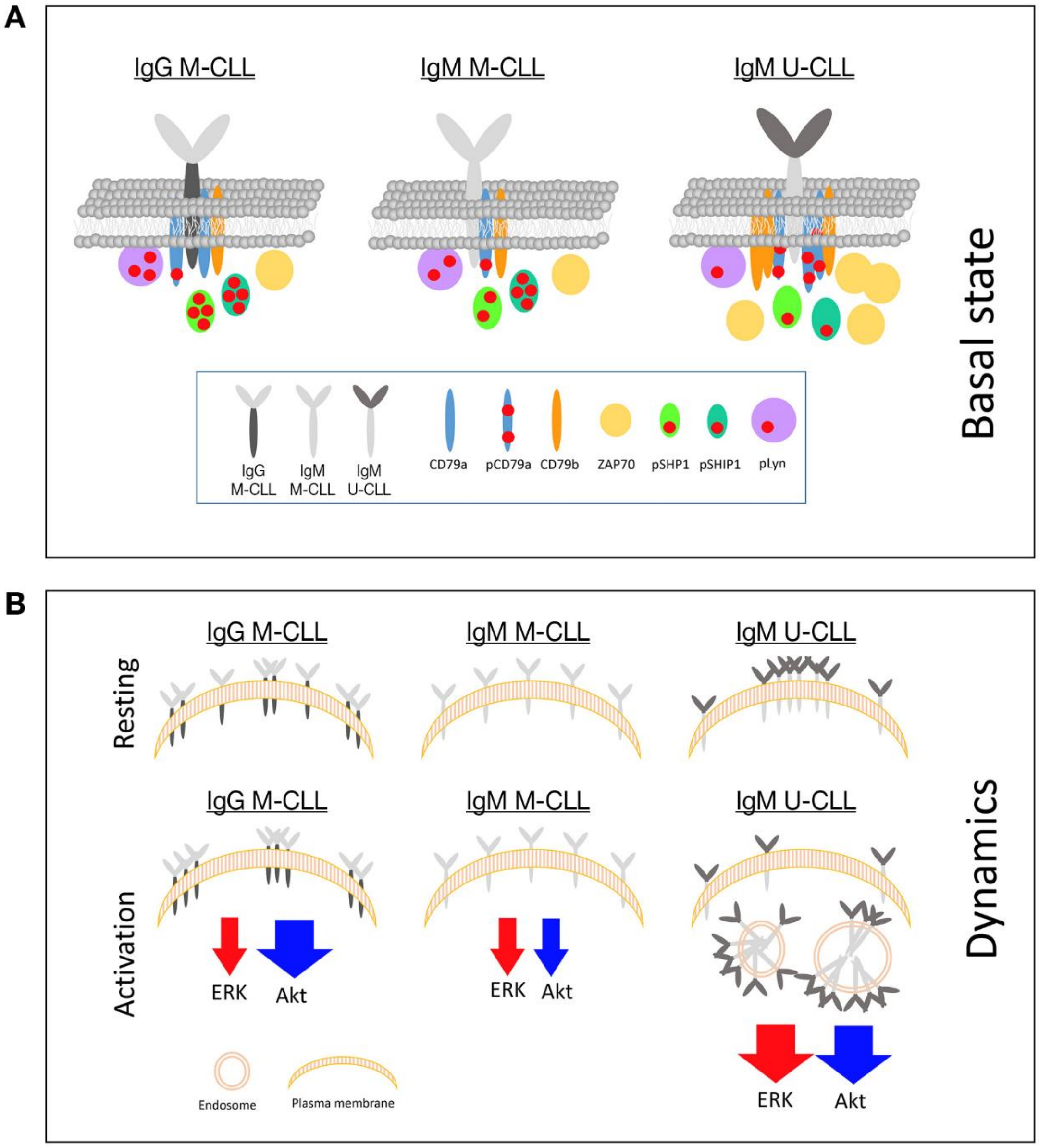

Supplementary Figure S7:The differences in basal BcR signalosomes and BcR spatial dynamics between immunogenetic CLL subtypes. A. Basal signalosomes compositions of IgG M-CLL, IgM M-CLL and IgM U-CLL are illustrated according to the molecules depicted in the insert. Small red circles indicate phosphorylation. B. The dynamics of IgG M-CLL (left), IgM M-CLL (middle) and U-CLL (right) is depicted in the resting (top) versus the activation (bottom) states of the BcR. Strength of the ERK (red) and Akt (blue) responses are indicated by the arrows width.

Supplementary Table 1: Patient characteristics.

**Patient**	Gender/Age (y)	ALC(x10^9/L)	WBC	IGHV gene SHM status
**CLL_01**	M/75	163.6	169.4	M-CLL
**CLL_02**	M/51	63.7	67.7	IgG M-CLL
**CLL_03**	F/73	81.8	94.3	M-CLL
**CLL_04**	F/86	42.1	48.8	IgG M-CLL
**CLL_05**	F/67	104.8	113	IgG M-CLL
**CLL_06**	M/86	81.8	88.3	U-CLL
**CLL_07**	M/60	215.7	219.5	U-CLL
**CLL_08**	F/72	233.7	253.9	U-CLL
**CLL_09**	M/71	41.7	47	M-CLL
**CLL_10**	F/68	124.6	135	M-CLL
**CLL_11**	M/63	103.3	124.9	M-CLL
**CLL_12**	F/70	130	138.1	IgG M-CLL
**CLL_13**	M/64	146.7	157.4	U-CLL
**CLL_14**	M/71	47.9	56.3	U-CLL
**CLL_15**	F/69	83.1	103.3	U-CLL
**CLL_16**	F/59	176.5	188.7	U-CLL
**CLL_17**	F/72	45.9	52.5	M-CLL
**CLL_18**	F/72	126.1	134.1	M-CLL
**CLL_19**	F/82	30.4	38.2	IgG M-CLL
**CLL_20**	F/72	97.9	106.7	IgG M-CLL
**CLL_21**	F/62	153.6	178	U-CLL
**CLL_22**	M/64	235.3	249	U-CLL
**CLL_23**	M/54	47.1	56.2	U-CLL
**CLL_24**	F/70	67.1	75.2	M-CLL
**CLL_25**	F/75	117.8	150.3	M-CLL
**CLL_26**	F/69	28.6	40.9	U-CLL
**CLL_27**	M/68	133	143.8	U-CLL
**CLL_28**	F/41	41.8	48.4	U-CLL
**CLL_29**	M/58	215.8	229	U-CLL
**CLL_30**	F/49	165.7	176.8	IgG M-CLL
**CLL_31**	M/82	21.6	25.9	M-CLL
**CLL_32**	M/71	94.1	97.9	U-CLL
**CLL_33**	M/52	137.1	147.1	U-CLL
**CLL_34**	M/52	126.8	134.8	M-CLL
**CLL_35**	M/81	84	89.4	U-CLL
**CLL_36**	F/52	130	141.1	U-CLL
**CLL_37**	M/72	30.9	38.5	M-CLL
**CLL_38**	M/51	31.9	37.5	M-CLL
**CLL_39**	F/86	99.3	124	U-CLL
**CLL_40**	F/74	42.6	48.6	M-CLL
**CLL_41**	M/50	80.3	91.9	U-CLL
**CLL_42**	M/73	103.5	110.1	U-CLL
**CLL_43**	M/70	100.6	106.1	U-CLL
**CLL_44**	F/66	171.9	186.2	M-CLL

**CLL_45**	M/84	98.7	135.7	M-CLL
**CLL_46**	M/52	50.9	54.6	M-CLL
**CLL_47**	M/76	81	89	M-CLL
**CLL_48**	M/75	81.3	86.4	M-CLL
**CLL_49**	M/80	68	84	U-CLL
**CLL_50**	M/57	71.4	76.6	U-CLL
**CLL_51**	M/70	12.7	18.5	U-CLL
**CLL_52**	F/55	99.5	109	IgG M-CLL
**CLL_53**	F/81	229.5	259	IgG M-CLL
**CLL_54**	M/68	43	46	IgG M-CLL
**CLL_55**	M/73	32.1	42	IgG M-CLL
**CLL_56**	M/66	12	26	IgG M-CLL
**CLL_57**	F/54	27.3	34.6	IgG M-CLL
**CLL_58**	F/76	200.9	210.2	U-CLL
**CLL_59**	M/67	75.6	81.9	U-CLL
**Normal _01**	F/27			
**Normal _02**	M/64			

M-male, F-female, y- years, ALC- absolute lymphocyte count, M-CLL: CLL with mutated IGHV genes; U-CLL: CLL with unmutated IGHV genes.

Supplementary Tables 2-4: Gene Set Enrichment Analysis for immunoglobulin subtypes in CLL .Gene Set Enrichment Analysis (GSEA) was used to identify overrepresentation of gene sets from the online database available at the GSEA Web site (http://www.broadinstitute.org/gsea/). In addition, select gene sets from the gene expression database of the Staudt laboratory (http://lymphochip.nih.gov/signaturedb/index.html) were uploaded to GSEA for inclusion in the analysis. Enriched or overrepresented gene sets between IgM U-CLL, IgM M-CLL and IgG M-CLL were identified using 1000 permutations of the phenotype labels.

Table 2. IgM U-CLL vs. IgM M-CLL		
	**NES**	**FDR q-val**
NF-κB signaling pathway	2.83	<0.0001
B-cell receptor signaling	2.39	<0.0001
TGF-β signaling pathways	2.18	<0.0001
Inflammatory response	2.17	<0.0001
Apoptosis regulation	2.1	<0.0001
TLR signaling pathway	1.91	0.003
IFN-γ response	1.81	0.002
RAS signaling pathway	1.77	0.003
IFN-α response	1.76	0.002
P53 signaling Pathway	1.75	0.002

Table 3. IgM U-CLL vs. IgG M-CLL		
	**NES**	**FDR q-val**
NF-κB signaling pathway	2.8	<0.0001
B-cell receptor signaling	2.17	<0.0001
TGF-β signaling pathways	2.11	<0.0001
Apoptosis regulation	1.92	<0.0001
Inflammatory response	1.8	0.006
IL4 response	1.73	0.008
P53 signaling Pathway	1.62	0.016
JAK/STAT3 signaling pathway	1.64	0.017
Ras signaling pathway	1.6	0.015
MYD88 signaling pathway	1.59	0.015

Table 4. IgG M-CLL vs. IgM M-CLL		
	**NES**	**FDR q-val**
P53 signaling Pathway	1.79	0.02
IFN-γ response	1.73	0.014
